# Respiratory Health Effects Associated with Restoration Work in Post-Hurricane Katrina New Orleans

**DOI:** 10.1155/2012/462478

**Published:** 2012-12-09

**Authors:** Roy J. Rando, John J. Lefante, Laurie M. Freyder, Robert N. Jones

**Affiliations:** ^1^Department of Global Environmental Health Sciences, Tulane School of Public Health and Tropical Medicine, 1440 Canal Street, Suite 2100, New Orleans, LA 70112-2704, USA; ^2^Department of Biostatistics and Bioinformatics, Tulane School of Public Health and Tropical Medicine, 1440 Canal Street, New Orleans, LA 70112, USA; ^3^Section of Pulmonary and Critical Care Medicine, Tulane School of Medicine, 1430 Tulane Avenue, New Orleans, LA 70112, USA

## Abstract

*Background*. This study examines prevalence of respiratory conditions in New Orleans-area restoration workers after Hurricane Katrina. *Methods*. Between 2007 and 2010, spirometry and respiratory health and occupational questionnaire were administered to 791 New Orleans-area adults who mostly worked in the building construction and maintenance trades or custodial services. The associations between restoration work hours and lung function and prevalence of respiratory symptoms were examined by multiple linear regression, *χ* ^2^, or multiple logistic regression. *Results*. 74% of participants performed post-Katrina restoration work (median time: 620 hours). Symptoms reported include episodes of transient fever/cough (29%), sinus symptoms (48%), pneumonia (3.7%), and new onset asthma (4.5%). Prevalence rate ratios for post-Katrina sinus symptoms (PRR = 1.3; CI: 1.1, 1.7) and fever and cough (PRR = 1.7; CI: 1.3, 2.4) were significantly elevated overall for those who did restoration work and prevalence increased with restoration work hours. Prevalence rate ratios with restoration work were also elevated for new onset asthma (PRR = 2.2; CI: 0.8, 6.2) and pneumonia (PRR = 1.3; CI: 0.5, 3.2) but were not statistically significant. Overall, lung function was slightly depressed but was not significantly different between those with and without restoration work exposure. *Conclusions*. Post-Katrina restoration work is associated with moderate adverse effects on respiratory health, including sinusitis and toxic pneumonitis.

## 1. Introduction

In August of 2005, Hurricane Katrina devastated the New Orleans area with high wind, heavy rainfall, and a storm surge of about 7 m which caused the collapse of the levee system surrounding the city. Approximately 80% of the city was flooded to varying depths for many weeks before the US Army Corps of Engineers was able to implement temporary levee repairs and install emergency pumping capacity.

In the aftermath of the flood event, the infrastructures of the city along with residences and commercial buildings were grossly contaminated with sediments deposited by the floodwaters and subsequently by microbial overgrowth supported by the residual moisture, high humidity, and elevated temperatures in the area. After floodwaters had receded, various surveys were conducted for measurement of indicators of microbial contamination in air, dust, and damaged building materials, including total and culturable mold spores, fungal fragments, mycotoxins, 1→3-*β*-D-glucan, and bacterial endotoxin. Generally, observed levels of microbial contaminants in these surveys were elevated, often extremely so, and were relatable to the depth and duration of flooding, and indoor levels were typically higher than those in the surrounding outdoor environment [1–5]. 

Subsequent to the posthurricane flooding event, there has been extensive rebuilding in the New Orleans area. Residents who personally performed repairs of their properties as well as various skilled and unskilled laborers working in the construction and building maintenance trades were at risk for inhalation exposures to dust containing microbial and other agents during demolition, removal, and repair of flood-damaged and contaminated infrastructure and building materials [6–8]. Exposures to microbial contaminants in agriculture, waste management, and in water-damaged and moldy buildings have been linked to various upper and lower respiratory illnesses and adverse effects including rhinitis, hayfever, toxic pneumonitis (TP), hypersensitivity pneumonitis (HP), and respiratory infections including pneumonia and exacerbation or initiation of asthma [9–12]. The potential for respiratory illness arising from inhalation exposure to bioaerosols and microbial contaminants during restoration activities in the post-Hurricane Katrina environment was of particular concern. As a part of a 5-year longitudinal study investigating the risk of respiratory illness associated with work in and around flood-damaged structures in post-Hurricane Katrina New Orleans, baseline findings from initial cross-sectional survey are reported. 

## 2. Methods and Materials

The study cohort consisted of 791 adults residing or working in the greater New Orleans metropolitan area. Study participants were recruited from several sources: (1) employees of three large institutions in the City of New Orleans, two of which are academic and the third is a branch of local government (*n* = 488 total). All three institutions experienced heavy flood damage to their buildings and facilities and utilized their regular staff as well as contract labor to perform restoration work. Recruiting from the academic institutions focused primarily on workers from departments normally engaged in maintenance, custodial, and facilities services. Support personnel (clerical, managerial, etc.) from the targeted departments were included in the recruitment. (2) Members of a local union hall for the skilled and unskilled building trades (*n* = 63). (3) Private building contractors and self-employed tradesmen (*n* = 95). (4) Other residents of the New Orleans area (*n* = 145), many of whom performed restoration work on their own properties. 

Overall, 54% of the study cohort reported a skilled or unskilled trade as their primary occupation, including carpentry (*n* = 50), electrician (*n* = 27), plumbing (*n* = 12), paint/drywall (*n* = 21), HVAC (*n* = 12), groundskeeping (*n* = 20), general construction (*n* = 102), general maintenance (*n* = 36), operating/building engineering (*n* = 18), and being mechanic/machinist (*n* = 15). An additional 15% of study participants worked in custodial or janitorial services (*n* = 115).

Testing was conducted in a mobile laboratory van outfitted with spirometry and interview work stations and ancillary equipment. The mobile laboratory van was moved to the work locations or union hall of the study participants for the duration of their respective testing period, generally 2 to 3 weeks, and to the parking lots of several large building supply stores, in order to allow private contractors and self-employed construction tradesmen to participate. 

Spirometry testing procedures and equipment have been previously described [13] and comply with both the original [14] and updated [15] American Thoracic Society spirometric test criteria. Spirograms were collected with a SensorMedics Model 1022 dry rolling seal spirometer interfaced to a laptop computer running OMI Spirometry software version 5.05.9 (Occupational Marketing, Inc., Houston, TX). All spirometric testing was conducted by the same individual who is a member of the research staff and is a Certified Pulmonary Function Technician; in addition, all spirometric test results were quality assured and interpreted by senior study investigators. 

Predicted lung function parameters and lower limit of normal (LLN) lung function values for forced expiratory volume in one second (FEV1), forced vital capacity (FVC), and FEV1/FVC ratio were computed from predictive equations developed by Hankinson et al. [16]. Separate predictive equations were used for Caucasians, African Americans, and Latinos. Predicted values for study participants of Asian heritage were calculated using the equations for Caucasians. In addition to race, the predicted values were based on age, gender, and height. The LLN values were calculated by subtracting 1.645 SEE from the predicted values, where SEE was the standard error of the estimate and 1.645 is the 95th percentile of a standard normal distribution.

Those participants with chronic obstructive pulmonary disease (COPD) were identified according to the GOLD criteria, that is, FEV1/FVC % predicted less than 70% and FEV1% predicted less than 80% [17]; however, only prebronchodilator lung function values were available and thus may not have adequately differentiated asthma (with reversible obstruction) from COPD. Those reporting “ever asthma” on questionnaire were therefore excluded from the analyses of COPD prevalence as a function of exposure.

A demographic, medical, smoking, and occupational questionnaire was administered to the study participants during the interview. It was based on a modified version of the standardized questionnaire reported by Burrows et al. [18], which accounts for a variety of putative and established risk factors and potential confounders for the development of airways disease including asthma, allergic disease, historical confounding exposures, serious childhood respiratory illness, cigarette smoking history, environmental tobacco smoke, and age, gender, and race. Additional questions were designed to capture the development of specific symptoms after Hurricane Katrina that might be associated with living and working in the post-Katrina environment. These included post-Hurricane Katrina onset of asthma, sinus symptoms, pneumonia, and transient fever and cough absent infection, with the latter used as an indicator of possible hypersensitive (HP) or toxic (TP) reaction. 

Asthma was defined dichotomously and required a positive response to both of the following questions: “Have you ever had asthma or attacks of shortness of breath with wheezing in the chest when not having a cold?” followed by “Do you still have asthma/ASOB?” The response to the question “How old were you when your asthma started?” was used in conjunction with the participant's date of birth to determine whether asthma onset was after September 30, 2005 (post-Katrina new onset asthma). Dyspnea was also defined dichotomously and required a positive response to the question “Do you have shortness of breath when hurrying on level ground?”

 The interview also included queries on pre- and post-Katrina work and occupation, and detailed information was gathered on time spent after Hurricane Katrina performing five specific types of hurricane/flood remediation work: demolition and ripout, trash removal, landscape restoration, sewer line repair, and mold remediation. Participation in any of these work activities, herein identified as “restoration work,” was assumed to result in occupational or vocational exposure to flood-related contaminants. Participants were asked to report the number of hours spent in each of the five restoration work activities, for each year since the hurricane up to the point of interview, and the type and relative frequency of any respiratory protective equipment that may have been used during the work. Restoration of personal property was included in the total time spent in restoration work along with any from the subject's regular employment.

The study protocol was approved by the authors' institutional review board and all study participants provided a written informed consent.

### 2.1. Statistical Analyses

Parallel questions for several symptoms were included in the interview. For the current hayfever compared to current trouble with pollen, grass, or fur, *χ* ^2^ analysis indicated that the parallel question significantly enhanced the positive response rate for these symptoms (22% claiming current hayfever versus 39% claiming sensitivity to pollen, grass, or animal fur; *P* < 0.0001). Similar but nonsignificant results were observed for ever and current asthma versus attacks of dyspnea and for chronic bronchitis versus COPD. 

The unadjusted prevalence rate ratios [19] for each symptom or condition for those doing any restoration work versus those not doing any restoration work were calculated within smoking categories based on 2 × 2 contingency tables. The prevalence rate ratio was defined as PRR = *p*_1_/*p*_2_, where *p*_1_ = *a*/*n*_1_ and *p*_2_ = *c*/*n*_2_ represent the sample proportion of exposed (*n*_1_) and unexposed (*n*_2_) individuals with disease. If *a* and *b* represent the number of exposed subjects who do and do not have disease, respectively, and *c* and *d* represent the number of unexposed subjects who do and do not have disease, respectively, the asymptotic 95% confidence interval for prevalence rate ratio is calculated using the following standard logarithmic transformation: $\ln\left(\text{PRR}\right)\pm 1.96\sqrt{b/an_{1}+d/cn_{2}}$. Exponential transformations on the confidence limits of this log transformed interval provided the asymptotic 95% confidence intervals for prevalence rate ratio [20]. 

Multiple logistic regression analyses were used to compute adjusted prevalence odds ratios [19] for each symptom or condition per 100 hours of restoration work as well as to compute asymptotic 95% confidence intervals for prevalence odds ratios. Due to significant interactions between gender and total hours of restoration work, logistic regression analyses were performed separately by gender and adjusted for age (because of a significant correlation with prevalence of pneumonia) and smoking categories. All interactions between age, smoking category, use of respiratory protective equipment (ever versus never), and total hours of restoration work were considered and were not significant. Multiple linear regression related %P FEV1, FVC, and FEV1/FVC to restoration work hours, use of respiratory protection, gender, asthma classification, and smoking category. All possible interactions were considered and were not significant, and no significant exposure associations were detected. All data analyses were performed using SAS 9.1.

## 3. Results

The majority of the study cohort was African American and male (Table 1). Hispanics accounted for 11% of the cohort. Current smokers comprised 28% of the cohort, whereas 18% were ex-smokers and 54% had never smoked. Upper and lower respiratory symptoms were prevalent in the cohort. 3.7% of the study cohort reported having pneumonia after Hurricane Katrina and almost half reported newly developing sinus symptoms. Among those reporting never having had asthma prior to Hurricane Katrina (*n* = 539), about 4.5% reported new onset asthma. Episodes of transient fever and cough occurring after Hurricane Katrina were reported by about 29% of the study cohort. Multiple episodes were also common in this reporting group: the median number of episodes was 3, and 10% of the group reported having 12 or more such occurrences.

Overall, lung function parameters were somewhat depressed in the cohort (Tables 1 and 4) and correlated with cigarette smoking and presence of current asthma symptoms. Percent predicted (%P) FEV1 averaged 93.4% (SD: 16.0) for current smokers, while ex- and never smokers had a mean level of 96.0% (SD: 15.4), *P* = 0.037 by *t*-test. The proportions of the cohort falling below LLN for FEV1 and FVC were also somewhat elevated (5% being the expected proportion based on the definition of LLN), particularly for the current and ex-smokers, as expected. Participants reporting current asthma symptoms had a mean %P FEV1 of 89.2% (SD: 21.8) and %P FVC of 91.8% (SD: 16.8), while asthmatics without current symptoms had a mean %P FEV1 of 95.8% (SD: 18.0) and %P FVC of 96.7% (SD: 15.2). Participants who never had asthma had mean %P FEV1 of 96.1% (SD: 13.6) and %P FVC of 96.4% (SD: 13.6).

 Almost 75% of the study participants reported having performed some restoration work activity after Hurricane Katrina (*n* = 587), and details on the actual time spent in these activities were self-reported by 474 or 81% of this group (Table 2). Demolition/ripout was the most commonly reported restoration work activity, followed by landscape restoration and trash/debris removal. Few study participants reported time spent in sewer line repair. Mold remediation was performed by about 21% of the study cohort. The distributions of time spent in these activities were highly skewed because many study subjects worked as much as 16 hours per day, seven days per week, for extended durations after Hurricane Katrina. The majority of the study subjects also reported time spent in more than one type of restoration work activity. For the total combined hours spent in any of the specific restoration work activities, the mean and median values reported by 474 subjects with complete data were 1646 and 620 hours, respectively. Among those who reported performing restoration work, 80.1% reported some use of respiratory protective equipment (i.e., filtering facepiece or air-purifying cartridge respirator). 202 study subjects reported no time spent in restoration work. 

The prevalence rates for post-Hurricane Katrina episodes of transient fever and cough and new onset sinusitis were significantly elevated for those reporting any restoration work (PRR: 1.7 and 1.3, resp.; Table 3). The prevalence rate ratios were statistically significant for ex- and never smokers but not current smokers. The prevalence rate ratios for post-Hurricane Katrina new onset asthma were elevated for the overall cohort (PRR = 2.2) and especially for ex- and never smokers (PRR = 2.7) but were not statistically significant (*P* = 0.09); the overall lack of significance may have been due in part to the low incidence (29 cases) and the reduced size of the base population; that is, only those who never had asthma prior to Hurricane Katrina. Statistically significant elevations in prevalence rate ratios for those having done any restoration work were not observed for pneumonia, dyspnea, COPD, and being below LLN for any of the lung function parameters.

**Figure 1:**
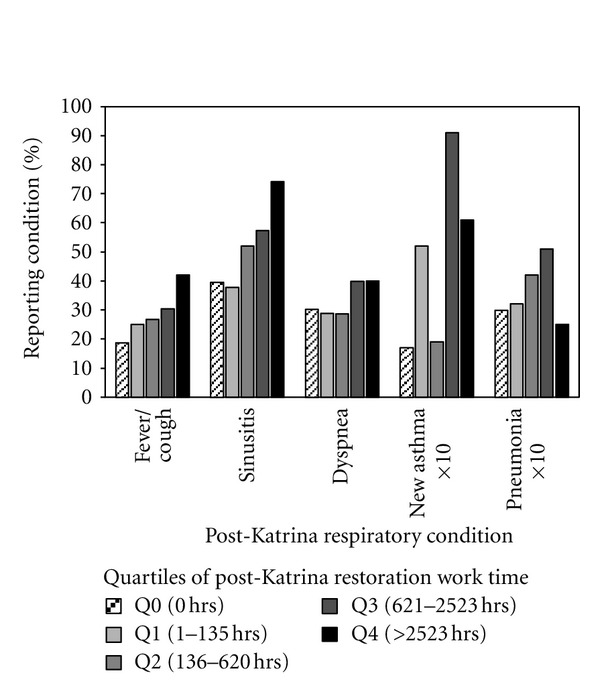
Respiratory symptom prevalence (unadjusted) within quartiles of post-Hurricane Katrina restoration work time. Quartiles are based on study subjects (*n* = 474) reporting detailed time spent in demolition, trash/debris removal, landscape restoration, sewer repair, or mold remediation. Q0 represents those who reported no time spent in restoration work activities (*n* = 202). Actual proportions with new onset asthma and with pneumonia are multiplied by 10 for graphical scaling and enhanced visualization of the data.

Figure 1 illustrates the prevalence of respiratory symptoms and conditions with quartiles of reported time spent in post-Hurricane Katrina restoration work activities (proportions for new onset asthma and pneumonia are multiplied by 10 in the figure for purposes of scaling). The unadjusted proportions for several post-Hurricane Katrina symptoms and conditions, including transient fever and cough, dyspnea, new onset sinus symptoms, new onset asthma, and pneumonia, show trends of increasing prevalence with time in restoration work. Statistically significant increases in prevalence odds ratio with restoration work time were observed only for transient fever and cough, for new onset sinus symptoms, and for dyspnea. When analyzed by logistic regression, the prevalence of fever and cough was statistically significantly associated with restoration work time, but only for men, with a prevalence odds ratio of 1.016 per 100 hours. Likewise, for new onset sinus symptoms in men, the prevalence odds ratio was 1.042 per 100 hours. In contrast, only women exhibited a statistically significant association between the prevalence of dyspnea and restoration work time, the odds ratio being 1.031 per 100 hours. The restoration work time-gender interactions observed in the logistic regression analysis of the prevalence odds ratios for these symptoms may be confounded with job type since women in the study tended to be in the custodial/janitorial occupations, whereas men were more likely to be in the building, construction, or maintenance trades. The exposures associated with restoration work done within these two broad categories of occupation are likely to be qualitatively and quantitatively different.

The prevalence odds ratios for post-Katrina new onset asthma and for pneumonia with restoration work time were elevated but were not statistically significant by logistic regression analysis. For new onset asthma the crude, unadjusted proportions in each of the restoration work time quartiles (Figure 1) were all higher than for participants with no restoration work time (1.9–9.1% versus 1.7%). For pneumonia, the unadjusted prevalence among those with no restoration work was 3.0% whereas subjects in each of the first three quartiles of restoration work time exhibited higher prevalence rates (3.2–5.1%); however, those in the highest quartile of restoration work time had a pneumonia prevalence rate of only 2.5%.

 Lung function measurements for the participants are summarized in Table 4. The group mean values for %P FEV1, FVC, and FEV1/FVC ratios generally were depressed but were within 5% of the normal except for smokers performing restoration work. Current smokers who did restoration work showed lower overall predicted lung function compared to smokers who did not; however, multiple linear regression analysis yielded no statistically significant correlations of any of the lung function parameters with restoration work time after adjustment for smoking, gender, asthma status, and use of respiratory protective equipment. 

## 4. Discussion

The results of this study suggest moderate adverse impact on respiratory health from time spent in post-Hurricane Katrina flood restoration activities. Published reports and public health surveillance systems generally did not show increases in emergency room visits or hospitalizations resulting from exposures in the post-Hurricane Katrina environment [21], although there have been a few reports of increased risk for respiratory effects. 

In a survey of 525 New Orleans firefighters [22], 79% had contact with floodwaters following Hurricane Katrina, and 38% reported new onset respiratory symptoms including sinus congestion, throat irritation, and cough. The prevalence rate ratio for those who had contact with floodwater versus those who did not was 1.9 and was statistically significant. First responders would have had significant exposures to flood sediments and associated contaminants, in addition to microbial agents. Inhalation exposure to aerosolized sediment collected in the aftermath of Hurricane Katrina was also shown to elicit significant pulmonary inflammation, increased airways resistance, and airway hyperreactivity in a mouse model [23].

There were widespread anecdotal reports of persistent nonproductive cough, often with sore throat and rhinorrhea, in the population residing in New Orleans in the Fall of 2005. This symptom complex became known as the Katrina cough. An investigation of this phenomenon by the Louisiana Department of Health and Hospitals [24] concluded that visits to medical facilities for respiratory complaints in the population of New Orleans were not related to exposure to dust or molds at the residence or at work. It is likely that Katrina cough was an irritant phenomenon resulting from a dry fall season with high levels of airborne particulate matter, coinciding with the start of the regular allergy and flu seasons [21, 24].

The prevalence of episodes of fever and cough in the present study population is clearly elevated for those who have done restoration work. Some of these cases may be relatable to Katrina cough. However, given the overall strong correlation with restoration work time, the common reports of multiple and distinct episodes of fever and cough, and the inclusion of the febrile component in the symptom complex, TP is likely to be underlying many of these reports and appears to be a common adverse effect of restoration work exposures in the post-Hurricane Katrina environment.

Unlike hypersensitivity pneumonitis, it is uncertain whether toxic pneumonitis and inhalation fevers result in significant lasting decrements in lung function, and functional parameters are expected to return to baseline upon recovery from an episode [25, 26]. This study did not identify any restoration work-related decrements in functional parameters, nor in the prevalence of being below LLN. The World Health Organization, in its report on guidelines on indoor air quality related to damp indoor spaces and mold [11], concluded that the evidence is inadequate to identify an association between damp indoor environments or the presence of mold with risk of alterations in lung function. However, in a recent study of 6,443 individuals in the European Community Respiratory Health Survey, lung function measurements across 9 years showed statistically significant excess declines in FEV1 of −2.25 mL/year and an additional −7.43 mL/year for women who reported dampness in the home and visible damp spots in the bedroom, respectively [27]. Annual excess declines of such small magnitude are difficult to detect over a short time period and are unlikely to result in a detectable group difference in function, measured cross-sectionally, after only a few years in the post-Hurricane Katrina environment, as in this baseline study. However, our study population is being evaluated annually over the course of a 5-year period, and currently undetectable decrements in lung function may reach the level of significance when measured directly over this extended period of time.

It is generally accepted that exposure to flood-related microbial contaminants can exacerbate existing asthma, and there is a mounting evidence that such exposures increase the risk of development of new asthma [11, 12]. In a recent extensive review and meta-analysis of the literature from 1980 to 2010 [12], overall odds ratios of 1.49 (C.I.: 1.28–1.72) and 1.68 (C.I.: 1.48–1.90) were found for the associations of asthma and wheezing, respectively, in children living in homes with visible mold. In this study, there was an observable elevation in prevalence of new onset asthma after Hurricane Katrina which increased with increasing quartiles of restoration work time but was not statistically significant. The lack of significance may be due in part to a self-selection process occurring in the cohort, with some study subjects who developed new onset asthma in the wake of Hurricane Katrina avoiding or terminating further restoration work exposure because of personal health concerns. Furthermore, the reported restoration work time is at the time of the interview, not at the time of development of new onset asthma, which always preceded the interview. Some of the study subjects may have continued to engage in and increase their time in restoration work to varying extent after developing asthma, but there is no information on the magnitude of this confounding nor its effect on the analysis.

This study has several additional limitations: as noted, health outcomes were assessed cross-sectionally, and there was a significant time element over which the baseline information was collected for the entire study population. Those subjects evaluated later in the study period could therefore have opportunity for greater amounts of time spent in restoration work with concomitant increase in risk for development of respiratory effects. As the longitudinal component of the study moves to completion, additional reports of development of respiratory symptoms and conditions are coming to light, and the ability to detect ongoing small excess decrements in lung function will also increase. Exposure to flood-related contaminants was assumed to be related to reported time spent performing restoration work. However, there is no information as to a particular individual's exposure intensity nor can it be assumed that all exposures associated with restoration work activities were qualitatively or quantitatively similar. Finally, the reliance on self-reporting of respiratory symptoms and conditions could have led to misclassification due to recall bias.

## 5. Conclusions

This study provides further evidence that workers performing restoration work on flood-damaged structures are at risk of respiratory health impacts from exposure to microbial-contaminated dust and debris. Moderate adverse respiratory health effects including toxic pneumonitis and sinusitis were commonly reported in the study cohort, and the prevalence of new onset asthma among restoration workers was noticeably elevated. While it is unclear from this cross-sectional analysis whether restoration work exposures have adversely affected pulmonary function in the population, the functional parameters overall are depressed in the cohort. Ongoing longitudinal health surveillance of this study cohort, along with a quantitative exposure assessment, will examine whether there is an increased risk for long-term or irreversible effects on respiratory health and how the risks relate to the nature and magnitude of the exposures occurring during posthurricane flood restoration work.

## Figures and Tables

**Table 1:** Demographics, symptoms, and respiratory conditions of the study cohort by smoking status.

	Smoking status			
	Current	Ex	Never	Overall
	(*n* = 218)	(*n* = 142)	(*n* = 431)	(*n* = 791)
Age: mean (S.E.)	43.2 (0.9)	51.8 (0.9)	43.5 (0.7)	44.9 (0.5)
% male	72.0	79.6	65.0	69.6
% non-white	73.8	64.8	77.1	75.0
% with any restoration work^a^	76.8	81.7	70.4	74.2
% cough	49.1	31.7	29.1	35.1
% phlegm	46.8	30.3	28.8	34.1
% dyspnea	35.8	41.6	31.6	34.6
% hayfever	21.6	25.0	22.0	22.4
% new onset sinus symptoms	51.7	51.1	45.7	48.4
(*n*_base_)^b^	(147)	(90)	(267)	(504)
% fever and cough	32.4	29.0	27.8	29.3
% ever asthma	19.7	20.4	21.6	20.9
% new onset asthma	2.8	4.0	5.4	4.5
(*n*_base_)^b^	(144)	(99)	(296)	(539)
% ever pneumonia	17.1	18.3	11.0	14.0
% new onset pneumonia	5.5	4.2	2.6	3.7
% COPD	9.7	6.5	3.6	5.7
% < LLN, FEV1	14.6	16.7	5.7	10.1
% < LLN, FVC	11.7	18.1	8.3	11.0
% < LLN, FEV1/FVC	8.7	6.5	3.6	5.5

LLN: lower limit of normal; FEV1: forced expiratory volume-1 second; FVC: forced vital capacity.

^a^Restoration work includes the following activities: demolition or ripout, trash or debris removal, landscape restoration, sewer line repair, and mold remediation.

^b^Excludes those reporting the symptom prior to Hurricane Katrina.

**Table 2:** Time spent performing restoration work Post-Hurricane Katrina for the study cohort, New Orleans, 2005–2010.

Restoration work activity	*n *	Hours of post-Hurricane Katrina restoration work				
		Mean	Range	25% tile	Median	75% tile
Demolition/ripout	391	1031	1–9384	87	480	1300
Trash removal	215	588	1–10800	32	167	725
Landscape restoration	226	601	1–7851	26	112	494
Sewer line repair	89	962	3–7851	30	173	1024
Mold remediation	160	322	1–4320	16	60	304
Any restoration work activity combined	474	1646	1–11750	135	620	2523

**Table 3:** Respiratory symptoms and conditions in the study cohort with restoration work by smoking status, post-Hurricane Katrina New Orleans, 2005–2010.

Smoking status	Univariate prevalence rate ratio (PRR): any restoration work versus none (95% confidence interval)								
	Fever and cough	New onset sinus symptoms	Pneumonia	New onset asthma	Dyspnea	COPD	FEV1 below LLN	FVC below LLN	FEV1/FVC below LLN
Current smokers	1.3 (0.8, 2.1) *n* = 210	1.1 (0.8, 1.6) *n* = 144	0.9 (0.3, 3.2) *n* = 214	1.0 (0.1, 9.1) *n* = 172	1.3 (0.8, 2.0) *n* = 215	2.8 (0.7, 11.6) *n* = 203	1.5 (0.6, 3.8) *n* = 203	1.2 (0.5, 3.0) *n* = 203	2.5 (0.6, 10.4) *n* = 203
Ex- & never smokers	2.0* (1.3, 3.0) *n* = 557	1.4* (1.1, 1.9) *n* = 354	1.6 (0.5, 5.4) *n* = 563	2.7 (0.8, 8.8) *n* = 461	1.2 (0.9, 1.5) *n* = 567	0.5 (0.2, 1.1) *n* = 554	0.7 (0.4, 1.3) *n* = 554	0.8 (0.5, 1.4) *n* = 554	0.6 (0.3, 1.4) *n* = 554
Overall	1.7* (1.3, 2.4) *n* = 767	1.3* (1.1, 1.7) *n* = 498	1.3 (0.5, 3.2) *n* = 777	2.2 (0.8, 6.2) *n* = 633	1.2 (1.0, 1.5) *n* = 782	0.9 (0.5, 1.8) *n* = 757	0.9 (0.6, 1.5) *n* = 757	0.9 (0.6, 1.4) *n* = 757	0.9 (0.5, 1.9) *n* = 757

*P < 0.05.

PRR: prevalence rate ratio; COPD: chronic obstructive pulmonary disease; LLN: lower limit of normal.

**Table 4:** Study cohort group averages for % predicted pulmonary function parameters, by smoking status, and performance of restoration work, post-Hurricane Katrina New Orleans, 2005–2010.

% predicted lung function -mean (S.E.)-	Current smokers: restoration work after Katrina? (*n*)		Ex- & never smokers: restoration work after Katrina? (*n*)	
	No (48)	Yes (155)	No (148)	Yes (406)
% P FEV1	95.3 (1.8)	92.7 (1.4)	95.0 (1.3)	96.4 (0.7)
% P FVC	96.2 (1.8)	94.9 (1.2)	94.9 (1.3)	95.1 (0.7)
% P FEV1/FVC	98.9 (1.0)	97.2 (0.8)	99.7 (0.7)	101.1 (0.4)

S.E.: standard error of the mean; %P FEV1: %predicted forced expiratory volume-1 second; %P FVC: %predicted forced vital capacity; %P FEV1/FVC: %predicted FEV1-FVC-ratio.
